# Delayed entry to care by men with HIV infection in Kumasi, Ghana

**DOI:** 10.11604/pamj.2015.22.107.7010

**Published:** 2015-10-07

**Authors:** Natasha Kumar, Rebecca Reece, Betty Norman, Awewura Kwara, Timothy Flanigan, Aadia Rana

**Affiliations:** 1 Warren Alpert Medical School, Brown University, Providence, RI, USA; 2 Department of Medicine, The Miriam Hospital, Providence, RI USA; 3 Komfo Anokye Teaching Hospital, Kwame Nkrumah University of Science and Technology, Kumasi, Ghana

**Keywords:** Gender disparities, men, HIV, Ghana, access to care, entry to care

## Abstract

**Introduction:**

In resource-limited settings, men may face considerable barriers to accessing HIV care as early interventions tend to focus on antenatal care settings.

**Methods:**

We performed a retrospective chart review of all adult patients referred to Komfo Anokye Teaching Hospital HIV clinic in Kumasi, Ghana in 2011 to identify the differences in clinical and demographic variables by gender at presentation to care using two-sample t tests with adjusted variance and Wilcox rank sum tests for continuous variables and Pearson chi-squared tests for categorical variables. We also compared differences in clinical and demographic variables among men stratified by CD4 count at initiation of care in order to identify variables associated with later entry to care.

**Results:**

Demographically, men were more likely to be older (men age 42 vs. 37, p<0.01), have a greater number of dependent children (1.8 v. 1.5, p = 0.04), to be living with or married to their partner (65.4% vs. 49.0%, p<0.01), and to have a higher level of education (tertiary education, 45.8% vs. 25.4%, p<0.01) than women. Clinically, men were more likely to have a lower CD4 count at entry to care (260 v. 311 cells/µL, p<0.01), to report clinical symptoms to the nurse during intake (p<0.01), and to have any history of alcohol use (p<0.01).

**Conclusion:**

Men in Ghana are accessing treatment at a later stage of their disease than women. Efforts to test and link men to care early should be intensified.

## Introduction

In resource limited settings (RLS), men have been identified as an at-risk population for delayed entry to HIV care, initiation of antiretroviral therapy (ART) and retention in care [1–7]. In sub-Saharan Africa, the focus of HIV treatment interventions has been on women, who are considered to be vulnerable to HIV infection due to biological factors and socio cultural factors that limit their sexual autonomy and power [1]. Recently, poorer health outcomes have been associated with male gender in the African HIV epidemic [1–5, 7]. This phenomenon has been connected to two causal factors: 1) the international focus upon interventions for prevention of maternal to child transmission (PMTCT) and 2) cultural values surrounding masculinity in sub-Saharan Africa that emphasize the invulnerability of men to illness [8]. Currently, there are few published studies on gender disparities in entry to HIV care in Western Africa. Nonetheless, national epidemiological data casts some light upon the gender dynamics of the HIV epidemic in Ghana. The Ghanaian HIV epidemic is predominantly driven by heterosexual transmission, which is responsible for 80% of new cases [9]. In 2011, of the 225,478 people living with HIV in Ghana, 100,336 (44.5%) were men and 125,141 (55.5%) were women [10]. However, 66.9% of people participating in anti-retroviral therapy in Ghana were women and only 33.1% were men. This indicates that 50% of women who need ART are engaging in treatment but only 39% of men who need ART are accessing it. Gender disparities have also been demonstrated in access to HIV testing. In Ghana, 21% of HIV-positive women have been found to utilize HIV counseling and testing, whereas only 14% of men utilize similar services [11]. In 2009, among adults aged 15-49, 6.8% of women had received an HIV test in the past 12 months and knew their results whereas only 4.1% of men had [10]. Assessments of existing gender disparities in access to care could provide evidence for interventions targeted toward men, a critical subpopulation implicated in the spread of the HIV epidemic in Ghana. While existing data shows gender disparities existing in the HIV epidemic, this study provides informative data on differences in immune status and other competing variables (e.g. occupation, alcohol use) that could play a role in gender disparities in entry to care or treatment adherence. In this retrospective chart review, we sought to identify the differences in clinical and demographic variables between men and women at presentation to care. We also sought to identify differences in clinical and demographic variables between men, stratified by CD4 count at presentation to care. The results of this study could serve as the basis for future in-depth research and intervention development to improve access to care for HIV-infected men.

## Methods

We performed a retrospective chart review of all adult HIV-positive patients entering care at Komfo Anokye Teaching Hospital in Kumasi, Ghana from January to December 2011. Komfo Anokye Teaching Hospital is a tertiary referral center that serves as the referral center for 6 of the 10 regions of Ghana. It is the second largest teaching hospital. The HIV clinic has over 5,000 patients, with over 50% of them on ART. Data Collection: Medical records at the hospital's HIV clinic are organized chronologically; the chart number contains the year in which the patient initiated care at the hospital. We utilized the chart numbers to identify all adult patients initiating care in 2011 and systematically reviewed the identified charts. Charts were excluded from the review if 1) the patient was below age 18 or 2) the form for the initial clinic visit indicated the patient had previous ART experience. We abstracted data on gender, age, marital status, occupation, education level, religion, number of dependent children, method of referral to HIV testing and treatment (physician-directed, voluntary, or PMTCT), funding method, experience with alcohol use, disclosure of HIV status, clinical assessment of WHO stage and CD4 count at presentation, and clinical symptoms. Our outcome of interest was stage of disease at presentation to care, as indicated by CD4 count, WHO stage and clinical symptoms upon presentation. Data Analysis: Descriptive analysis was performed on the individual variables with means for continuous variables and percentages for categorical values. Differences in abstracted variables between men and womenwere analyzed using two-sample t tests with adjusted variance and Wilcox rank-sum tests for continuous variables and using Pearson chi-squared tests for categorical variables. Differences in abstracted variables between subgroups of men stratified by CD4 count (CD4<100, 100-199, 200-349, 350-499, >500) were also assessed. Ethics Approval: This project was approved by the Institutional Review Board at Brown University in Providence, RI and the Committee on Human Research, Publications, and Ethics at Kwame Nkrumah University of Science and Technology, School of Medical Sciences and Komfo Anokye Teaching Hospital in Kumasi, Ghana.

## Results

Of the 1,127 patients who initiated care during the study period, 895 patient charts were included in the review of which 279 (31.2%) were men and 616 (68.8%) were women. Of the 232 patients excluded from the study, 156 charts were missing, and 76 charts were excluded because 63 were treatment-experienced, 9 had blank charts, and 4 were younger than 18 years old. The baseline characteristics of the study population by gender are summarized in Table 1. The mean (standard deviation, SD) age of women was 37 (10.4) years and the mean age of men was 42 (9.3) years. The majority of patients were married (44.4% of women and 63.2% of men). Additionally, 18.5% of women were divorced and 15.1% of men were single. Trading was the most popular occupation for both genders (61.3% of women and 22.4% of men). Most men (38.7%) had some high school education, whereas women were either uneducated (24.8%) or had received some middle school education (26.6%). Most patients (76.4%) had dependent children and 81.8% of patients identified themselves as Christians. Overall, 51% of men versus 44.6% of women had an initial CD4 count below 200. More than half of patients (59.1%) complained of clinical symptoms to the intake nurse during their initial visit. Statistically significant differences between genders were observed for several variables (Table 2). Demographically, men were more likely to be older (men age 42 vs. 37, p<0.01), have a greater number of dependent children (1.8 v. 1.5, p = 0.04), to be living with or married to their partner (65.4% vs. 49.0%, p<0.01), and to have a higher level of education (tertiary education, 45.8% vs. 25.4%, p<0.01) than women. Clinically, men were more likely to have a lower CD4 count at entry to care (260 v. 311 cells/µL, p<0.01), to report clinical symptoms to the nurse during intake (p<0.01), and to have any history of alcohol use (p<0.01). There was no statistically significant difference between genders in WHO stage at entry to care (p = 0.60) or between the proportion of men (19.4%, n = 54) and women (19.6%, n = 121) who did not return after HIV testing at their initial visit for a CD4 count assessment (p = 0.92). Men with lower CD4 counts (Table 3) were more likely to be referred to the clinic based on diagnostic HIV testing rather than voluntary HIV testing (p<0.01) and to present with physical exam findings (p = 0.03) or report symptoms to the physician (p = 0.03) at their initial visit than men with higher CD4 counts; there were no statistically significant differences in demographic variables between men when stratified by CD4 count.

**Table 1:** Overview of demographic and clinical variables by gender

Variable	All (N = 895)	Female (N = 661)	Male (N = 279)
	n (%)	n (%)	n (%)
Mean (SD) age (years)	39 (10.3)	37 (10.4)	42 (9.5)
Marital status			
Single	115 (13.8%)	76 (13.2%)	39 (15.1%)
Married or cohabiting	419 (50.2%)	278 (49.0%)	168 (65.4%)
Divorced	134 (16.0%)	107 (18.5%)	27 (10.5%)
Widowed	95 (11.4%)	80 (13.9%)	15 (5.8%)
Separated	34 (4.1%)	26 (4.5%)	8 (3.1%)
Occupations			
Unemployed	46 (5.6%)	36 (6.4%)	10 (3.9%)
Trading	404 (49.3%)	347 (61.3%)	57 (22.6%)
Driving	36 (4.4%)	0 (0.0%)	36 (14.3%)
Farming	72 (8.8%)	44 (7.8%)	28 (11.1%)
Masonry	16 (2.0%)	0 (0%)	16 (6.3%)
Teaching	18 (2.2%)	10 (1.8%)	8 (3.2%)
Hairdressing	36 (4.4%)	33 (5.8%)	3 (1.2%)
Student	15 (1.8%)	12 (2.1%)	3 (1.2%)
Seamstress	36 (4.4%)	27 (4.8%)	11 (4.4%)
Other	137 (16.8%)	57 (10.1%)	80 (31.7%)
Educational level			
Primary	255 (31.1%)	201 (40.1%)	54 (24.0%)
Secondary	241 (33.2%)	173 (34.5%)	68 (30.2%)
Tertiary	230 (31.7%)	127 (25.4%)	103 (45.8%)
Has a religious affiliation	779 (96.5%)	547 (97.7%)	232 (92.8%)
Mean number of dependent children	1.6	1.5	1.8
History of alcohol use	68 (16.6%)	27 (11.6%)	41 (38.0%)
Referral Method			
Diagnostic	617 (81.0%)	414 (78.4%)	203 (86.8%)
Voluntary	103 (13.5%)	72 (13.6%)	31 (13.2%)
PMTCT	42 (5.5%)	42 (8.0%)	0 (0.0%)
Reported symptoms to nurse at during intake	466 (59.1%)	302 (55.6%)	164 (67.0%)
Reported symptoms to physician at initial visit	279 (78.4%)	195 (80.2%)	84 (74.3%)
Physical exam findings present at initial visit	159 (41.8%)	107 (42.0%)	52 (41.6%)
Mean CD4 count	296	311	260
Non pregnant women		395	
Pregnant women		303	
Missing CD4 count	175 (19.6%)	121 (19.6%)	54 (19.4%)
Mean WHO stage	2.78	2.77	2.79

**Table 2:** Gender differences in clinical and demographic variables

Variable	Female	Male	P Value
**Mean (SD) age (years)**	37 (10.4)	42 (9.5)	<0.01
**Marital status**			
Married or cohabiting	278 (49.0%)	168 (65.4%)	<0.01
**Educational level**			<0.01
Primary	201 (40.1%)	54 (24.0%)	
Secondary	173 (34.5%)	68 (30.2%)	
Tertiary	127 (25.4%)	103 (45.8%)	
**Has a religious affiliation**	547 (97.7%)	232 (92.8%)	<0.01
**Mean number of dependent children**	1.5	1.8	0.04
**History of alcohol use**	27 (11.6%)	41 (38.0%)	<0.01
**Reported symptoms to nurse during intake**	302 (55.6%)	164 (67.0%)	<0.01
**Mean CD4 count**	311	260	<0.01

**Table 3:** Differences in clinical and demographic variables among men, by CD4 count

Variable	Men, Stratified by CD4 Count					P Value
** CD4 Count:**	**<100**	**100-199**	**200-349**	**350-499**	**>500**	
					
	**(N = 79)**	**(N = 39)**	**(N = 44)**	**(N = 33)**	**(N = 32)**	
**Mean (SD) age (years)**	42 (8.9)	42 (9.7)	43 (8.5)	40 (9.9)	41 (9.4)	0.86
**Marital status**						
Married or cohabiting	53 (68.8%)	18 (52.9%)	31 (72.0%)	22 (71.0%)	21 (70.0%)	0.40
**Educational level**						0.95
** **Primary	17 (24.6%)	6 (20.7%)	12 (30.0%)	4 (14.3%)	6 (25.0%)	
** **Secondary	20 (29.0%)	10 (34.5%)	11 (27.5%)	9 (32.1%)	7 (29.2%)	
** **Tertiary	32 (46.3%)	13 (44.8%)	17 (42.5%)	15 (53.6%)	11 (45.8%)	
**Has a religious affiliation**	63 (87.5%)	29 (90.6%)	42 (97.6%)	31 (96.9%)	28 (93.3%)	0.27
**Mean number of dependent children**	1.8	1.9	1.6	1.9	1.8	0.93
**Alcohol use**	15 (33.3%)	8 (36.4%)	11 (39.3%)	4 (66.6%)	2 (40.0%)	0.63
**Referral method**						<0.01
** **Diagnostic	68 (95.8%)	30 (90.9%)	34 (87.2%)	20 (76.9%)	20 (71.4%)	
** **Voluntary	3 (4.2%)	3 (9.1%)	5 (12.8%)	6 (23.1%)	8 (28.6%)	
**Reported symptoms to physician at initial visit**	28 (54.9%)	10 (41.7%)	6 (18.8%)	4 (40.0%)	2 (33.3%)	0.03
**Physical exam findings present at initial visit**	28(56.0%)	10(20.0%)	6(12.0%)	4(8.0%)	2(4.0%)	0.03

## Discussion

While several researchers have concluded that men are at risk for delayed engagement and poor retention in HIV care in resource-limited settings [1–8], the study of gender disparities in HIV care has not yet extended to healthcare settings in Ghana or even in the West African region, as defined by the Economic Community of Western Africa (ECOWAS). Our review of patients’ CD4 count, WHO stage and clinical symptoms at presentation to care in Ghana found that men had a lower CD4 count and reported more clinical symptoms at presentation to care than women did. This aligns with other studies conducted in other countries in Africa, which found that men were at risk for delayed entry to care [2–8]. Men's poorer immune status can also be correlated to the existing epidemiologic data on HIV testing and treatment in Ghana, which found that a smaller proportion of HIV-positive men were getting tested or treated for their disease compared to HIV-positive women.

Our outcome of interest was stage of disease at presentation to care, as indicated by CD4 count, WHO stage and clinical symptoms upon presentation. Men presented to care with an average CD4 count of 260 cells/µL, whereas women presented with an average CD4 count of 311 cells/µL (p = 0.03), a difference of 51 cells/µL. Many studies have found clinically significant gender disparities in initial CD4 count [5, 7]. In our study, men also were more likely to report at least one of five common clinical symptoms (cough, diarrhea, dysphagia, rash or jaundice) to nurses at their initial visit (p = 0.003). While this finding goes hand in hand with the CD4 count disparity between genders, few studies have measured clinical symptoms at presentation, except for one systematic review that included measurements of hemoglobin and assessed patients for anemia [6]. Our study did not identify a statistically significant difference between genders in WHO stage at presentation to care. Overall, 73% of patients presented to the clinic at an advanced stage of disease (WHO stage III or IV per clinical assessment), which is higher than the findings of several other studies, where 44.2-62% of patients in Sub-Saharan African populations presented to care at WHO stage III or IV [5–7]. Only one study, which assessed 55,789 patients in three Sub-Saharan countries, found a gender disparity in WHO stage at presentation [7].

Demographic variables measured within our study such as age, marital status and education have been correlated with delayed entry to HIV care within the literature. While our analysis shows no statistically significant differences between these demographic variables among men with high CD4 counts compared to men with low CD4 counts, we did find differences in these variables between men and women. Older age has been associated with delayed HIV testing and entry to care as well as decreased desire for HIV testing [12–15]. Being unmarried and living in a household alone have also been tied to late presentation to care [8], and men were more likely to be cohabiting or married than women in our study. Higher education levels have been tied to increased knowledge about HIV status and increased interest in HIV testing [15, 16], though our study found that men were more educated than women and delayed their engagement with HIV care.

We were unable to assess disparities in access to care based on either PMTCT interventions or men's migratory career choices due to a paucity of chart data on these variables, though both of these factors have been associated with gender disparities in engagement with HIV care within the literature. PMTCT has been identified as a vehicle for women's entry into care, particularly HIV testing [10, 17]. However, missing data limited our ability to assess the effect of PMTCT interventions on access to care. The initial CD4 counts at entry to care of women diagnosed through PMTCT interventions at the hospital were rarely found in the charts at the adult HIV clinic because pregnant women are first diagnosed and treated for HIV at the antenatal clinic and then transferred to the adult HIV clinic after giving birth. While the initial CD4 count of women diagnosed through PMTCT was in fact lower than non-pregnant women or men, the sample size was too small to conduct statistical analyses (n = 10, Table 1). Improvements in documentation and transfer of care as well as studies that span both the antenatal and adult HIV clinic would be necessary in order to better understand the effect of PMTCT interventions on access to care at this tertiary care center. Men's participation in migratory careers such as mining has been tied to increased risk of HIV infection as well as difficulty in engaging with HIV care [17, 18]. In our chart review, we found that men and women were equally likely to be employed in some fashion, but sample sizes within specific occupations were too small to adequately assess gender disparities by occupation (Table 1). There are several limitations to this analysis. The chart review utilized retrospective data from medical records. As a result, the findings allow for limited interpretation and causal factors cannot be determined. Additionally, a significant amount of 2011 data was missing (13.3% of charts, n = 156), though this proportion is similar to or much smaller than the proportion measured by other studies. As previously explained, initial CD4 counts for women entering HIV care through PMTCT interventions at this teaching hospital were often missing.

## Conclusion

This retrospective chart review of all patients entering HIV care at a tertiary hospital in Kumasi, Ghana showed that men are presenting with more advanced disease than women. Men account for 45.5% of HIV positive adults in Ghana but only 31.2% of HIV positive patients at a tertiary care center. Men may not be seeking care consistently in Ghana and are accessing treatment at a later stage of their disease than women. Further research needs to be aimed at improving factors impacting late diagnosis and access to care. Many interventions in Africa have targeted men's risky sexual behavior, i.e. primary prevention [19–21], but limited efforts have been made to improve treatment of HIV, i.e. secondary prevention, for this subpopulation. A small body of evidence is emerging that interventions integrated into the workplace and educational settings where men predominate may be effective in connecting men to care [22]. Efforts to test and link men to care early should be intensified.
